# Additive mixed modeling of impact of investment, labor, education and information technology on regional income disparity: An empirical analysis using the statistics Indonesia dataset

**DOI:** 10.1016/j.dib.2022.108619

**Published:** 2022-09-17

**Authors:** Regina Niken Wilantari, Syafira Latifah, Wahyu Wibowo, Harun Al Azies

**Affiliations:** a Department of Economics, Faculty of Economic and Business, University of Jember, 68121 Jember, Indonesia; b Department of Business Statistics, Faculty of Vocational Studies, Institut Teknologi Sepuluh Nopember, 60111 Surabaya, Indonesia; c Department of Statistics, Faculty of Science and Data Analytics, Institut Teknologi Sepuluh Nopember, 60111 Surabaya, Indonesia

**Keywords:** Additive mixed model, Education, Income disparity, Information technology, Investment

## Abstract

The data that is the subject of the case study in this article is secondary data in the form of panel data. Data from the Statistics Indonesia database and Central Bureau of Statistics database of each province on Java Island (DKI Jakarta, West Java, Central Java, DI. Yogyakarta, East Java, and Banten). This panel dataset consists of five research variables, namely a response variable which is the value of the income disparity index for each province in Java using the Williamson index calculation standard, and four predictor variables namely investment (GFCF), labor, the ICT Index, and education index of six provinces of Java Island, Indonesia during the period 2010 -2019.

## Specifications Table

**Table:** 

Subject	Economics
Specific subject area	Regional economics, Economic inequality
Type of data	Cross-sectional and panel data
How the data were acquired	Data Extracted from the Statistics Indonesia database listed on the website: https://www.bps.go.id
Data format	Raw and analyzed. In the “Experimental Design, Materials, and Methods” section below, we will explain the process of processing datasets.
Description of data collection	The data in this study includes information on income disparities, investment, labor force, science and technology, and education in six provinces of Java, Indonesia. Specifically for income disparity data, this data is processed data using the standard Williamson index calculation which will be explained in the “Data description” section below.
Data source location	Secondary data sources in six provinces (Banten, DKI Jakarta, West Java, Central Java, DI Yogyakarta and East Java) on the island of Java, Indonesia. The direct URL to the data is shown in Table 2. At the data source location, all provinces of Indonesia are available, but in this study only six provinces were used, so the rest provinces have been eliminated from the dataset until a suitable dataset has been obtained as in the “Data Accessibility” section.
Data accessibility	With the article. Data is in a Microsoft Excel file. Sheet 1 presents the raw data, and Sheet 2 explains the data label Repository name: Mendelay Data Data identification number: 10.17632/gvpmbd47hv.1 Direct URL to data: https://data.mendeley.com/datasets/gvpmbd47hv

## Value of the Data

• The uniqueness of this data is the use of the Williamson index for the income inequality indicator approach and the use of other indicators for household panels in Java, Indonesia.
• These data are useful for the general public to understand the influence of work, physical investment, technology, and education on income inequality. For researchers, this article is a research reference in the field of development economics, especially to calculate convergence between regions, and is quite possible in the expansion of statistical analysis. These data are also important for regulators, namely the government, in the development of policies that lead to a strategy for the development of the distribution of income between regions.
• The data set and articles will enable other researchers to replicate the current study and to conduct cross-regional convergence tests in the future.

## 1 Data Description

Data were collected from the database of the Central Bureau of Statistics of Indonesia and the database of the Central Bureau of Statistics of each province on the island of Java (DKI Jakarta, West Java, Central Java, DI Yogyakarta, East Java, and Banten) with five research variables, as for the operational definitions of the research variables used are presented in Table 1.

**Table 1:** Research variables.

Variable type	Indicator	Operational definition
Response Variable	Income disparity (IW)	Income disparity is the difference in per capita income that occurs between regions within an area [1] which is calculated using the Williamson Index [2]. The data used to calculate income inequality is GRDP per capita and population in 2010-2019 which comes from the Statistics Indonesia for 6 provinces in Java Island. The Williamson Index unit is expressed in ratios
Predictor Variable	Investment (GFCF)	Investing is the investment of certain funds at the moment to make profits in the future [3]. In this study, the data used is Gross Fixed Capital Formation (GFCF) where GFCF is a form of physical investment in the form of capital goods such as buildings, machinery, equipment, vehicles [4]. Data comes from the Statistics Indonesia for 2010-2019 in trillion rupiahs. The calculation of the GFCF can be done by direct or indirect methods, depending on the availability of data that can be obtained in each region. In this study, the direct approach is used, i.e. summing all the GFCF values that occur in each industry (business field). The data to directly calculate the GFCF can be obtained from the financial statements of each region. The available data includes fixed asset change information/data which is valued based on price (ADH) or purchase price (acquisition). To obtain the value of the GFCF at Constant Prices (ADHK) 2010, the GFCF ADHB is “deflated” (divided) by the wholesale trade price index (IHPB) according to the group of capital goods
	Labor	The labor force is the working-age (aged 15 years and over) population who are already working, looking for work, who are in school, and residents who take care of the household [5]. In this study, the calculation of the labor force is by dividing the number of people aged 15 years and over who work by the working age population, then multiplying the result by 100. The data used comes from the Statistics Indonesia in 2010-2019 as a percentage.
	ICT Index (ICT.Index)	Technology is the result of the development of science which can not only be a tool but also new skills and processes/methods. In this study, the data used is the Information and Communication Technology Development Index (IP-ICT) which is a standard measure that can describe the level of development of information and communication technologies in a region.
	Education Index (Edu.Index)	The education index is an indicator that facilitates the analysis of the problem of disparity of results in each variable of the education sector and facilitates the analysis of the overall performance of education results in an area. In this study, the calculation of the schooling index consists of dividing by two the results of the index of the school life expectancy and the mean years of schooling then multiplied by 100. The data used come from the Statistics Indonesia in 2010-2019 which are expressed as a percentage..

**Table 2:** Description location of the data source.

Indicator	Direct URL to data for each indicator
Investment (GFCF)	Direct URL to data for GFCF indicators [Part1] https://www.bps.go.id/publication/2015/10/30/bd0e19d80d21570116f42b5a/produk-domestik-regional-bruto-provinsi-provinsi-di-indonesia-menurut-pengeluaran-2010——-2014.html Direct URL to data for GFCF indicators [Part2] https://www.bps.go.id/publication/2020/04/30/25e3ca3836c003ffcaa1bacc/produk-domestik-regional-bruto-provinsi-provinsi-di-indonesia-menurut-pengeluaran–2015-2019.html

Labor	https://www.bps.go.id/statictable/2016/04/04/1907/penduduk-berumur-15-tahun-ke-atas-menurut-provinsi-dan-jenis-kegiatan-selama-seminggu-yang-lalu-2008—2022.html

ICT Index (ICT.Index)	Direct URL to data for ICT Index indicators [Part1] https://tinyurl.com/ICT-Index-Part-1 Direct URL to data for ICT Index indicators [Part2] https://tinyurl.com/ICT-Index-Part-2 Direct URL to data for ICT Index indicators [Part3] https://tinyurl.com/ICT-Index-Part-3 Direct URL to data for ICT Index indicators [Part4] https://tinyurl.com/ICT-Index-Part-4

Education Index (Edu.Index)	Direct URL to data for school life expectancy indicators https://www.bps.go.id/indicator/26/417/1/-metode-baru-harapan-lama-sekolah.html Direct URL to data for mean years of schooling indicators https://www.bps.go.id/indicator/26/415/1/-metode-baru-rata-rata-lama-sekolah.html

The Williamson index used by Jeffrey G Williamson in his 1965 study is a measure of income disparity. This Williamson index is the distribution coefficient of the average distribution value which is calculated based on the estimated value of the GDRP per capita and the population of the areas of the area analyzed [6]. In contrast to the Gini ratio to measure income distribution, the Williamson index uses the gross domestic product (GDP) per capita as a basis. This study uses the Williamson index because the method compares the level of income between regions, not the level of prosperity between groups [7]. The Williamson index is statistically formulated as follows

(1) $$IW=\frac{\sqrt{\sum_{i=1}^{n}{\left(y_{i}-y\right)}^{2}\left(\frac{f_{i}}{n}\right)}}{y},\;0<IW<1$$

With *y*_i_ is *i*-th regional GRDP per capita, meanwhile *y* is GRDP per capita on average for all regions. Total population of the *i*-th area is *fi* and *n* is total population of the whole area. The Williamson Index value ranges from zero to one (0-1). The smaller the number of the Williamson index, the smaller the disparity or vice versa, or in other words more unequal. The specifies the criteria used to determine the level of disparity with the following criteria in Table 3 [8].

**Table 3:** The disparity level criteria.

Cut of value (The Williamson Index)	Disparity level
0.0 to 0.2	Low disparity
0.21 to 0.35	Moderate disparity
> 0.35	High Disparity

This study measures regional income disparity using the Williamson index. The Williamson index, which is used to measure regional income inequalities, can be calculated using per capita GDRP data across regions, both GDP per capita at constant or current prices, as well as interregional demographic data. Table 4 below is a descriptive analysis to explain the results of the calculation of the Williamson index. Based on the calculation of the Williamson Index, it will be known how high-income disparity occurs in each province of the island of Java.

**Table 4:** Total population, GRDP per capita at constant 2010 prices, and Williamson's Index in Java Island by province in 2019

Province	Total population (Millions)	GRDP per capita (Thousands-IDR)	Williamson Index
DKI Jakarta	10,56	208,374.92	0.52
West Java	49,32	28,950.47	0.69
Central Java	34,72	28,984.00	0.66
DI Yogyakarta	3,84	30,108.90	0.47
East Java	39,7	41,901.60	0.97
Banten	12,93	47,753.87	0.63
**Java Island**	**150,4**	**56,191**	**0.66**

Table 4 shows that DKI Jakarta province is the area with the highest GRDP per capita, which is 208,374 IDR with an income disparity based on the Williamson index calculation of 0.52. East Java Province ranks second with GRDP based on the highest constant prices after DKI Jakarta Province, inversely proportional to the value of GRDP per capita of East Java Province is very low compared to GRDP per resident of DKI Jakarta province. This makes the level of disparity in the province of East Java be at the top of the island of Java, which is 0.97. Other information from Table 4, West Java Province as the area with the most populous population on the island of Java, although based on the value of GRDP, is included in the third-highest category after East Java province, but the per capita GRDP of West Java province also tends to be lower, followed by a high disparity rate of 0.69. Meanwhile, DI Yogyakarta Province, although it has the lowest per capita GRDP value in Java, its per capita GDP is almost close to the per capita GRDP value of Central Java and West Java provinces which have GRDP values nine times greater than the GRDP of DI Province of Yogyakarta. This condition proves that provinces with high levels of disparity tend to have low values of GDP per capita. High-income disparity occurs when in one region there is an imbalance in economic growth that causes some regions to have high GDRP values but not match the increase in GDRP in other regions. In addition, population density can also affect the high level of income disparity.

**Fig. 1:**
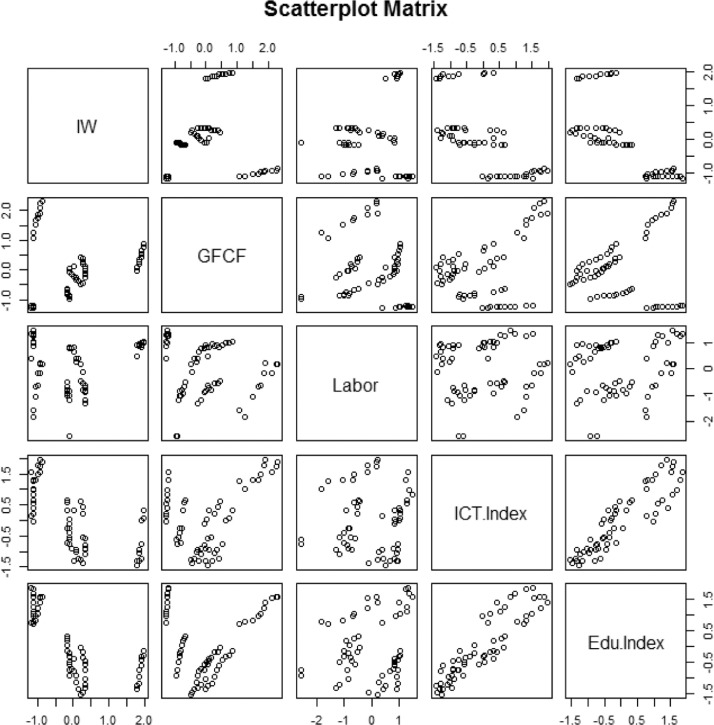
Scatter plot of regional income disparity and its predictor variables

The scatter plots reveal linearity or non-linearity between variables and are used to identify the type of relationship between variables [9]. Fig 1 describes the relationship between regional income disparity and predictor variables in general but does not exclusively explain linearity. Based on the scatter plot, it can be seen that the relationship between the regional income disparity variable and the GFCF and labor is visually plotted on the graph with a random distribution, meaning it has no strong relationship, this is also based on the low-value correlation between the regional income gap with the GFCF variable and labor which is not significant at the 95% level (Table 5). The correlation between regional income disparity with the technology index and the education index is negative and significant at a 95% confidence level (Table 5). The p-value 0.001 (<= 0.05) rejects H_0_.

**Table 5:** The correlation results

Parameter 1	Parameter 2	r	Confidence Interval 95%	P-value
Income Disparity	GFCF	0.093	[-0.16; 0.34]	0.4766
Income Disparity	Labor	0.157	[-0.10; 0.39]	0.2289
Income Disparity	ICT.Index	-0.659	[-0.78; -0.49]	0.001
Income Disparity	Edu.Index	-0.721	[-0.82; -0.57]	0.001

Based on the correlation test in Table 5, which is an analysis aimed at showing the direction and strength of the relationship between variables, the results are quite varied for the relationship between regional income disparities and the GFCF, labor, technology index, and education index. The technology index variable and the education index have a negative relationship with regional income disparities depending on the value of the correlation coefficient. This means that if both variables increase, it will lead to a decrease in regional income disparities for Java and vice versa. Based on the significance of the p-value, it was concluded that there was no relationship between the regional income disparity variable and the predictor variable GFCF, and the labor variable, with a value of positive but very weak correlation.

## 2 Experimental Design, Materials and Methods

Additive Mixed Models is one of the applications of Semiparametric Mixed Models, where the mixed model framework can be applied to a semiparametric regression model based on penalized splines [10]. The mixed model is very useful for analyzing data with a group data structure because it considers the effects of dependencies within the group [11]. Additive Mixed Models is a popular method to solve the problem of semiparametric clustered data [12]. The general form of Additive Mixed Models is as follows [13].

(2) $$y_{ij}\left(t\right)=\mu_{ij}\left(t\right)+\varepsilon_{ijt}$$

with $\mu_{ij}\left(t\right)$ are an unknown smooth regression function and an independent $\varepsilon_{ijt}$ error with zero mean and constant variance. The model equation (2) can be formulated by constructing $\mu_{ij}\left(t\right)$ in the following equation [14].

(3) $$y_{ij}\left(t\right)=U_{i}+\beta \text{subjec}t_{i}+f\left(x_{ij}\right)+\varepsilon_{ijt}$$

**Fig. 2:**
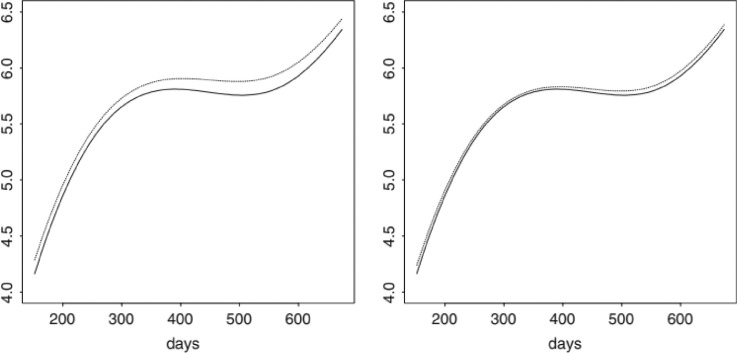
Various estimates of “*f*” for a pooled data set [15]

In Equation (3) where $y_{ij}$ is the logarithm of the *j-*th measure of the *i-*th subject, $U_{i}$ is the random intercept for the *ith* subject assuming that $U_{i}∼N\left(0,\sigma_{U}^{2}\right)\;$and $\varepsilon_{ijt}∼N\left(0,\sigma^{2}\right)$ are independent, subject-i is the dummy variable indicating the group that gathers the data with β being the subject-specific functional random intercept, $x_{ij}$ indicating the predictor variable where the measurement $y_{ij}$ is taken, and the smoothing function *f* modeled with a condemned regression spline, the illustration of the smooth function *f* is presented in Fig 2 below, which is a Sitka spruce growth dataset from [15].

Solid lines in both panels indicate the approximate *f* obtained by the fit model (3) to the original data set. The dotted line in the left pane indicates the weak estimate of *f* calculated from the modified data set, while the dotted line in the right pane shows the corresponding strong estimate. The idea of the additive mixed model is well illustrated using data on regional income disparity in Java Island, Indonesia. Using data from Statistics Indonesia with measurements during 2010-2019. The results of the additive mixed model between variables with the general form of the regional income disparity model on the island of Java with DKI Jakarta Province as the reference group are as follows.

(4) $$\begin{array}{ccc} IW_{ij} & = & U_{i}+f\left(GFCF_{ij}\right)+f\left(Labor_{ij}\right)+f\left(ICT.Index_{ij}\right)+f\left(Edu.Index_{ij}\right)\\ & & +\beta_{1}West\;Java_{i}+\beta_{2}Central\;Java_{i}+\beta_{3}DI\;Yogyakarta_{i}+\beta_{4}\;East\;Java_{i}\\ & & +\beta_{5}Banten_{i}+\varepsilon_{ij} \end{array}$$

In Eq. (4) where $IW_{ij}$ is the j-th income disparity measurement in the i-th province.

$U_{i}$ is intercept and $\beta_{1},\beta_{2},\beta_{3},\beta_{4}\&\beta_{5}$ is the mean differences in income disparity between other provinces and DKI Jakarta Province, meanwhile $\varepsilon_{ij}$ is model error. Based on Table 6 and implementing Eq. (2), the additive mixed model of regional income disparity in Java is obtained as follows

(5) $$\begin{array}{ccc} IW_{ij} & = & U_{i}+6.36\left(GFCF_{ij}\right)+0.99\left(Labor_{ij}\right)+1.00\left(ICT.Index_{ij}\right)+1.00\left(Edu.index_{ij}\right)\\ & & +1.593\;West\;Java_{i}+1.246\;Central\;Java_{i}-0.465\;DI\;Yogyakarta_{i}+3.120\;East\;Java_{i}\\ & & +0.733\;Banten_{i}+\varepsilon_{ij} \end{array}$$

**Table 6:** Model significance results

Approximate significance				
Variable	e.d.f	F-value	P-value	Confidence Interval 95%
f(GFCF)	6.360	21.627	0.000*	[-0.57; 0.26]
f(Labor)	0.999	0.197	0.659	[-0.03; 0.06]
f(ICT.Index)	1.000	1.876	0.178	[-0.06; 0.01]
f(Edu.Index)	1.000	0.419	0.521	[-0.05; 0.11]

Parametric coefficients				

Province	Coefficient	t-value	P-value	Confidence Interval 95%

West Java	1.593	20.195	0.000*	[1.44; 1.74]
Central Java	1.246	12.371	0.000*	[1.05; 1.44]
DI Yogyakarta	-0.465	-1.564	0.125	[-1.05; 0.13]
East Java	3.120	28.985	0.000*	[2.91; 3.33]
Banten	0.733	5.344	0.000*	[0.46; 1.00]
R^2^(adj)	0.998			

Note: *) Significant at α = 5%, effective degrees of freedom (e.d.f)

Based on the significant results, each variable has different results. Here are the results of the interpretation of the additive mixed model and an analysis of the effect of physical investment (GFCF), number of workers (Labor), technology index (ICT.Index), and education index (Edu.Index) on regional income inequalities in Indonesia, especially Java Island.

### 2.1 The effect of Gross Fixed Capital Formation (GFCF) on regional income disparity

**Fig. 3:**
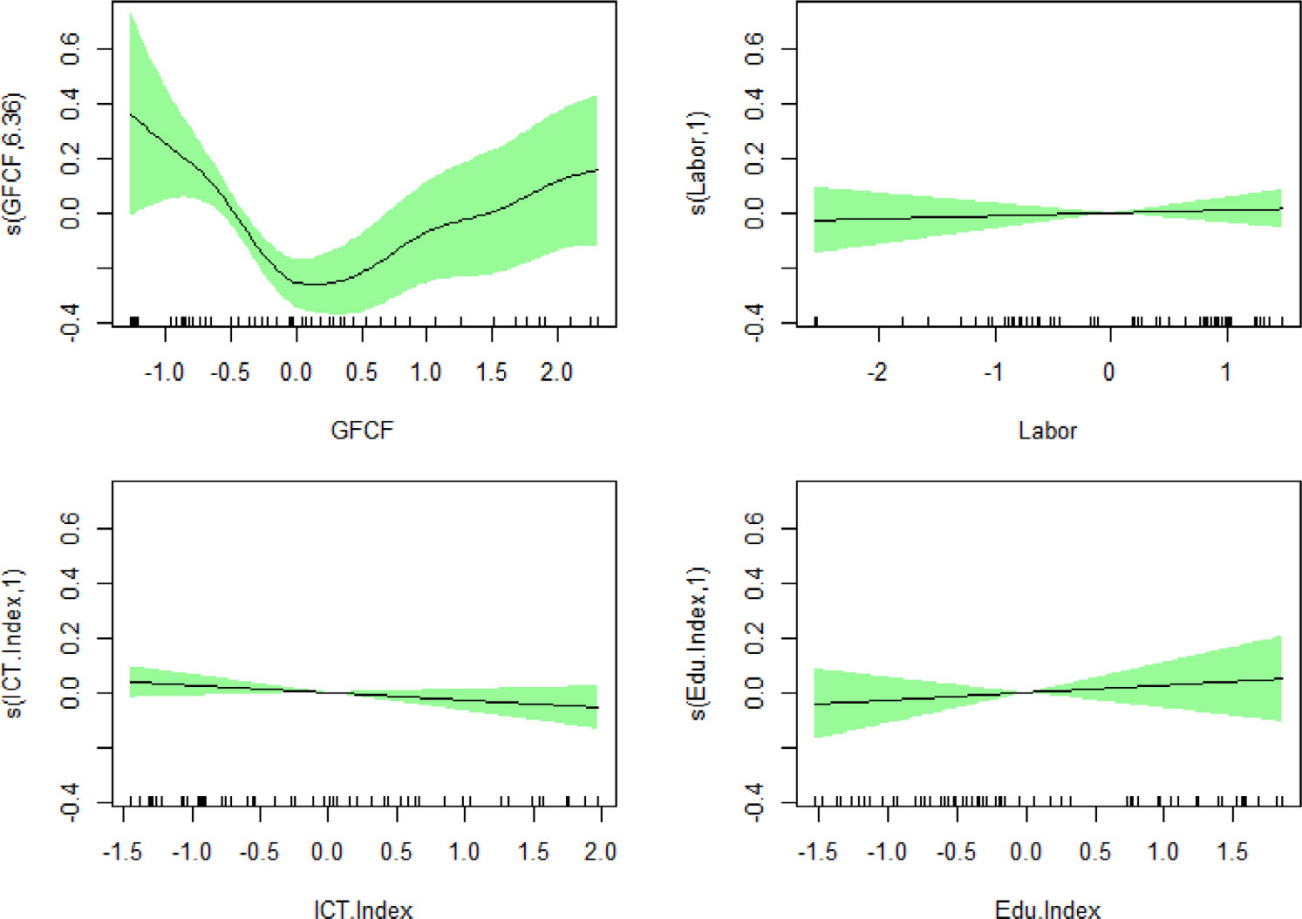
The fitted penalized spline for the predictor variable effect

The physical investment variable represented by the variable GFCF has an effective degrees of freedom (e.d.f) value of 6.36. The value of e.d.f shows the variation in the influence of the predictor variable on the response variable and shows linearity, the higher the value of e.d.f, the less linear. However, if e.d.f is equal to 1, it means linear. The GFCF variable has a nonlinear influence on the income disparity variable based on the value of e.d.f, in addition to this, it can also be seen from the shape of the produced GAM graph (Fig 3). The amount of GFCF produces the most varied effect on regional income disparity. The significance test shows that the GFCF has a significant effect on regional income disparities, although the GFCF has no relationship or relation to regional income disparities, as shown in the results of Table 5, but can influence regional income disparities. Since in the results of the correlation analysis (Table 5), the concept that the two variables used are symmetric [16], there is no difference between the response variable and the predictor variable, it does not, therefore, does not apply to the modeling results in Table 6 because the results in Table 6 distinguish the response from the predictor variable.

### 2.2 The effect of the number of the labor force on regional income disparity

The number of the labor force has no varying effect on regional income disparity with an e.d.f value of 0.999 and the basis of a significance test at a significance level of 5% with a confidence interval [-0.03; 0.06].

### 2.3 The effect of the technology index on regional income disparity

The technology index does not produce variable effects on regional income disparity, and statistically, the technology index has no effect on regional income disparity based on significance tests at a significance level of 5% with a confidence interval [-0.06; 0.01]. These results indicate that although the correlation analysis (Table 5) shows that the technology index has a relationship with regional income disparities, it does not necessarily affect regional income disparities

### 2.4 The effect of the education index on regional income disparity

The e.d.f value of the education index variable is 1.00, which shows the least variable effect on regional income disparity and a linear relationship because the e.d.f value is 1.00. Significance test based on the confidence interval [-0.05; 0.11] shows that the education index variable has no significant effect on regional income disparity. Based on these results, the results are consistent with the conclusions of the previous technology index which, by correlation analysis (Table 5), has a relationship with regional income disparities but shows no effect on regional income disparities.

### 2.5 Income disparity differences between DKI Jakarta and West Java provinces

The table above shows that the estimated 95% confidence interval for $\beta_{1}$ in Eq. (5) is (1.44; 1.74), indicating a statistically significant difference between DKI Jakarta province and the province of West Java in terms of regional average income disparity.

### 2.6 Income disparity differences between DKI Jakarta and Central Java provinces

The test results also explain that the regional income disparity for the province of Central Java with a 95% confidence interval for $\beta_{2}$ is (1.05; 1.44), which shows that there is a difference significant with the province of DKI Jakarta.

### 2.7 Income disparity differences between DKI Jakarta and DI Yogyakarta province

The results were different in DI Yogyakarta province, namely, there was no significant difference in regional income disparity between the DI Yogyakarta province and DKI Jakarta province based on the interval of 95% confidence for $\beta_{3}$ (-1.05; 0.13).

### 2.8 Income disparity differences between DKI Jakarta and East Java province

Based on the 95% confidence interval for $\beta_{4}$ (2.91; 3.33), which shows a statistically significant difference between DKI Jakarta Province and East Java Province about regional disparity revenues.

### 2.9 Income disparity differences between DKI Jakarta and Banten province

The significant difference between regional income disparity in Banten Province and DKI Jakarta Province based on the 95% confidence interval for $\beta_{5}\;$is (0.46; 1.00).

Table 6, in addition to summarizing the results of the significance of the model on the influence of predictor variables on regional income disparities, Table 6 also summarizes the results of the significance of the model in explaining the differences in disparities between provinces of Java. In this study, the results of the inter-provincial additive mixture model refer to the general form of the model (Equation 4) the regional income gap on the island of Java and the province which is used as a reference (comparison) is DKI Jakarta Province. This is based on the fact that DKI Jakarta province is the capital of the country with a high level of economic growth according to the Statistics Indonesia Dataset. Based on Table 6, the average regional income disparity in West Java, Central Java, East Java, and Banten provinces shows a significant difference with DKI Jakarta province. This means that there are differences in regional income disparities in the four regions with DKI province of Jakarta, while only the DI province of Yogyakarta has no differences in regional income disparities with DKI province of Jakarta. This finding is derived from the results of calculating the Williamson index in Table 3, which shows quite a significant difference in the value of the Williamson index between DKI Jakarta province and four other provinces, namely West Java, Java Central, East Java, and Banten.

The accuracy of the regional income disparity model which is influenced by GFCF and the difference with four other provinces (West Java, Central Java, East Java and Banten) in Java is 99.8%, so this model can be used as a benchmark to explain regional income disparity in Java.

In addition to the significance of the model, to determine the magnitude of the effect of the predictor variables, a penalty curve is installed for each predictor variable. Based on the graph Fig 3, the shaded areas correspond to the 95% confidence interval estimates. Note that GFCF shows the largest effect and has a nonlinear relationship because the value of e.d.f (Table 5) is the largest compared to the other variables, which is 6.360. In addition, the education index variable, the ICT index, and the labor force do not have a nonlinear effect because the resulting e.d.f value is 1

## Ethical Statement for Data in Brief

I testify on behalf of all co-authors that our article submitted to Data in Brief:

*Title:*

Additive Mixed Modeling of Impact of Investment, Labor, Education and Information Technology on Regional Income Disparity: An Empirical Analysis Using the Statistics Indonesia Dataset

*All authors:*

Regina Niken Wilantari, Syafira Latifah, Wahyu Wibowo, Harun Al Azies

*Corresponding author's email address:* e-mail: wahyu_w@statistika.its.ac.id

1. The authors of the original research report have presented an accurate account of the work carried out as well as an objective discussion of its significance;
2. The authors approved that the article published in formats for Data in Brief;
3. The authors may be asked to provide research data that supports the paper for editorial review and/or to meet the journal's open data requirements;
4. This material has not been published in whole or in part elsewhere;
5. The manuscript is not currently being considered for publication in another journal;
6. The authors have been personally and actively involved in substantive work leading to the manuscript, and will hold themselves jointly and individually responsible for its content;
7. The authors declare that they have no known competing financial interests or personal relationships that could have appeared to influence the work reported in this paper.

## CRediT Author Statement

**Regina Niken Wilantari:** Conceptualization, Methodology; **Syafira Latifah:** Data collection and curation; **Wahyu Wibowo:**: Resarch initiation, Funding, Validation; **Harun Al Azies:** Software, Writing and Editing.

## Declaration of competing interest

The authors declare that they have no known competing financial interests or personal relationships that could have appeared to influence the work reported in this paper.

## Data Availability

Disparity_dataset (Original data) (Mendeley Data).
